# Recent Advances in Genetic and Epigenetic Modulation of Animal Exposure to High Temperature

**DOI:** 10.3389/fgene.2020.00653

**Published:** 2020-07-07

**Authors:** Jiong Wu, Weiwei Zhang, Chenghua Li

**Affiliations:** ^1^State Key Laboratory for Managing Biotic and Chemical Threats to the Quality and Safety of Agro-products, Ningbo University, Ningbo, China; ^2^Laboratory for Marine Fisheries Science and Food Production Processes, Qingdao National Laboratory for Marine Science and Technology, Qingdao, China

**Keywords:** heat exposure, genetic mechanism, epigenetic regulation, immunity, heat acclimation

## Abstract

Animals have evolved multiple systems, including genetic and epigenetic systems, to respond accordingly to heat exposure and heat acclimation. Heat exposure greatly affects immunity, changes metabolic processes, and poses a serious threat to animals. Heat acclimation is induced by repeated organism exposure to heat stress to dissipate heat. This review focuses on genetic modulation via heat shock transcription factors and calcium as two important factors and compares the changes in HSPs under heat stress and heat acclimation. Epigenetic regulation summarizes the role of HSPs in DNA methylation and histone modifications under heat stress and heat acclimation. These genetic and epigenetic modifications protect cells from thermal damage by mediating the transcriptional levels of heat-responsive genes. This review highlights recent advances in the genetic and epigenetic control of animal thermal responses and their interactions.

## Introduction

Terrestrial and aquatic organisms are subjected to environmental stressors (abiotic and biotic factors), such as pathogen attacks and heat stress (Kultz, 2003; Norouzitallab et al., 2014). The intensity of thermal stress is expected to increase in the coming decades because of climate change (Ishita et al., 2010). When temperature is above the prescriptive zone of animals, their core body temperature increases, resulting in hyperthermia and altering various biological functions, such as breaking down biological regulatory mechanisms, deteriorating health, declining immunity, and increasing mortality (Logan and Buckley, 2015; Han et al., 2016; Nyboer and Chapman, 2017; Negrón-Pérez et al., 2019). Heat-shock response (HSR) was originally discovered as a transcriptional response to elevated temperature shock (Ritossa, 1996). Its discovery led to the identification of heat shock proteins and heat shock factor 1 (HSF1). Accumulating evidence shows that HSF1, the central player in HSR, is mediated according to specific cellular requirements through cell-autonomous and non-autonomous signals (Li et al., 2017). HSF1 enhances transcriptional activity, especially the molecular chaperones heat-shock protein family (HSP), which helps protect the structural stability of refolding or degrading intracellular proteins (Yura et al., 1993; Goldberg, 2003). Heat acclimation is induced by repeated organism exposure to heat stress to dissipate heat and reduce heat illness (Carter et al., 2005; Hung et al., 2005; Magalhães et al., 2010). In addition to mammals, scholars are also studying heat acclimation and thermal death in insects (Bowler and Hollingsworth, 1965; Hoffmann et al., 2003; Weldon et al., 2011; Allen et al., 2012). Results suggested that heat acclimation has an important relationship with HSR. Heat acclimation invokes HSR, whereas HSR promotes the development of heat acclimation (Yamada et al., 2007; Mcclung et al., 2008; Magalhães et al., 2010).

In addition, calcium, as the hallmark of heat acclimation, is an important second intracellular messenger that can convert extracellular stimuli into intracellular signals, as well as regulate cell development, survival, differentiation, and gene expression by affecting signaling pathways (Berna-Erro et al., 2012). Calcium and HSF1 also play a key role in the stress response of biological cells under heat stress, such as endoplasmic reticulum (ER) stress, reactive oxygen species (ROS) stress (Ryu et al., 2013), and apoptosis (Manucha et al., 2011). Therefore, in this review, we discussed the genetic mechanisms of HSR and heat acclimation mainly focusing on HSF1 and calcium. The core mechanisms HSF1, calcium, and critical genes involved in HSR are summarized.

Genetic regulation or environmental exposure alone often cannot fully explain the process of thermoregulation. Epigenetics is a regulatory system for environmental stress without change in the DNA arrangement, and it plays a vital role in genome stability, nuclear organization, transcription, and imprinting (Bartels et al., 2018). To date, various epigenetic modifications, including DNA methylation, histone modifications, and non-coding RNA, have been studied in which the transcription level, not the DNA sequence, is changed (Arányi et al., 2005; Huang and Fan, 2010). DNA methylation, which frequently occurs at the CpG dinucleotide in eukaryotes, is presented among the most extensively studied epigenetic regulatory mechanisms. Genome-wide methylation profiling has been recently conducted for economically important animals (Khavari et al., 2010; Almamun et al., 2014; Smallwood et al., 2014; Almamun et al., 2015). In particular, numerous genes undergo DNA methylation under heat stress, which affects the expression of many genes. Dynamic changes in DNA methylation present a specific pattern in tissues or cell types. Epigenetic mechanisms may play an important role in the formation of heat acclimation and the changes in mitochondrial respiratory chain (OXPHOS) (Dai et al., 2018). However, few studies have been conducted on the relationships between genetic and epigenetic regulations under heat stress. This review discusses several important physiological processes, briefly introduces the genetic and epigenetic mechanisms of animal responses to heat, and emphasizes the relationships between these mechanisms.

## Genes Modulate Animal Responses to Heat Stress

### Heat and Oxidative Stress

Previous studies have reported that heat stress induces ROS production, which, in turn, produces oxidative stress (Ryu et al., 2013). Two time-dependent oxygen free radicals have been identified under heat stress in human umbilical vein endothelial cells: O^2–^ and H_2_O_2_ (Li et al., 2014). Under heat stress, O^2–^ noticeably increases immediately, and then H_2_O_2_ increases, indicating that the generation of primary ROS mainly depends on excess O^2^ after heat stress.

The role of HSP genes under oxidative stress, the production of HSF1, is well known (Lee et al., 1999; Chuang et al., 2002). The role of small HSPs (sHSPs, molecular weight 8.5–40 kDa) and the HSP60, HSP70, and HSP90 protein families in protective stress response to heat stress has been studied. The HSP70 family abolishes heat-induced ROS production (Sreedhar et al., 2002; Belhadj et al., 2014). HSP70 suppresses mitochondrial damage by reducing ROS level in rat histiocytoma (Sreedhar et al., 2002). HSP90 reduces ROS damage by inhibiting ASK1-p38 activation induced by H_2_O_2_ in human umbilical vein endothelial cells (Zhang et al., 2005). HSP60, as a typical mitochondrial protein in eukaryotes, inhibits apoptosis through the outer mitochondrial membrane under heat stress (Song et al., 2018).

Certain sHSPs, such as HSPB1 (HSP25/27) and HSPB5 (αB-crystallin), are known to participate in oxidative stress reactions that reduce the level of oxidative damage by maintaining the redox state of cells (Mehlen et al., 1996). sHSP overexpression leads to increased antioxidant glutathione (GSH) concentration and increased glucose 6-phosphate dehydrogenase (G-6-P-DH) activity, which contribute to the formation of reduced GSH in murine L929 fibrosarcoma cells (Mehlen et al., 1996).

Under oxidative stress, HSF1 has been shown to synergize with nuclear factor erythroid 2-related factor 2 (Nrf2) to bind to promoters and transcriptionally upregulate several target gene expression, such as sequestosome-1 (SQSTM1, also known as p62) and heme oxygenase-1 (HMOX1) (Jain et al., 2010; Samarasinghe et al., 2014; Watanabe et al., 2016), activating transcription factor 3 (ATF3) (Kyu-Han et al., 2009; Takii et al., 2010), HSPA1A/B/L (HSP70), and HSPB1 (HSP25/27) (Mehlen et al., 1996). Therefore, HSF1 and HSP can induce a variety of cytoprotective mechanisms and protect related genes as a response to oxidative stress.

Moreover, heat stress increases the metabolic rate. The organism consumes more oxygen, which leads to electron leakage in the respiratory chain and an increase in ROS levels. Complex I and complex III is the main source of ROS; the former increases ROS through glutathione, the latter through ubisemiquinone radical intermediate (QH) at the Q_O_ site of complex III (Turrens, 1997; Julie et al., 2002; Muller et al., 2003; Taylor et al., 2003), while calcium stimulates Krebs cycle enzymes and oxidative phosphorylation in the mitochondria to promote ATP synthesis (Brookes, 2004; Görlach et al., 2015). Under normal circumstances, calcium reduces ROS from complexes I and III, and when these complexes are inhibited, calcium can enhance ROS production (Görlach et al., 2015). The three-dimensional conformational changes in the respiratory chain complex may be regulated by calcium to increase ROS level (Brookes, 2004). Although calcium plays an important role in affecting changes in ROS content, the specific mechanisms underlying this process have not been elucidated.

### Heat and Endoplasmic Reticulum Stress

The ER is a dynamic organelle whose functions include protein folding, calcium buffering, and lipid and carbohydrate metabolism (Song et al., 2018). Under heat stress, diverse cellular stresses, such as perturbations in calcium homeostasis, redox imbalance, and protein folding defects, cause misfolded and unfolded proteins to accumulate in the ER lumen, which triggers the unfolded protein response (UPR) (Qian et al., 2011; Zhang et al., 2014; Zhang S. et al., 2017). UPR activates the genes encoding the ER resident protein required for protein folding (Hetz, 2012). This regulatory response transmits signals from the ER lumen to the nucleus via the inositol-requiring protein 1α/spliced X-box binding protein 1 (IER1α/XBP1) by the conserved intracellular signaling pathway of UPR (Barna et al., 2018). The UPR signals through the sensor IRE1α, which controls the splicing of the mRNA encoding the transcription factor XBP1. Together with UPR, HSR protects eukaryotic cells from the damage caused by protein toxicity. Experiments have demonstrated that HSP72 (HSPA1A) and HSP90 (HSPC1), two HSF1-regulated chaperones, interact with IRE1α cytoplasmic domains to enhance the complex of IRE1α/XBP1 at the ER, and this process, in turn, affects UPR in cell culture (Marcu et al., 2003; Gupta et al., 2010; Ahmed and Averill-Bates, 2015). ATPase domain of Hsp72 binds to the monomeric and nonphosphorylated cytoplasmic tail of IRE1α, in order to protect IRE1α from ER stress-mediated phosphorylation and oligomerization of IRE1α. In addition, increased XBP1 protein is required for enhanced cell survival induced by Hsp72 under ER stress conditions. Furthermore, Hsp90 has been shown to increase the half-life of IRE1α by binding to the cytoplasmic domains. The molecular mechanisms by which HSP72 and HSP90 inhibits ER stress need more experiments to explore.

Aside from HSF1, calcium has been identified to be associated with ER stress. Under heat stress, the accumulation of unfolded proteins in the ER causes calcium to leak from the ER into the cytoplasm by Bak/Bax, then, causes the ER to induce apoptosis (Bhandary et al., 2012). The calcium releasing is mainly through redox sensitivity of IP3R. In addition, cytoplasmic calcium overload can lead to cytotoxicity, which can lead to the activation of endogenous or mitochondrial-dependent apoptotic pathways (Crompton et al., 2002).

### Heat and Apoptotic Pathway

The mitochondria are central integrators and transducers of pro-apoptotic signals (Crompton et al., 2002). Recent evidence suggests that the heat-shock proteins HSP90, HSP70, and sHSPs can inhibit heat-induced cell death by intervening at various steps of the mitochondrial-dependent apoptotic pathway (Roufayel and Kadry, 2019). Both sHSPs (HSP27) and HSP70 inhibit the release of cytochrome c and, consequently, the intrinsic pathway of apoptotic cell death in cell culture (Samali et al., 2001; Klein and Brüne, 2002; Sancho, 2003). Further experiments found that HSP70 and HSP90 may inhibit apoptosis through the caspase 3 pathway because ROS-mediated caspase 3 activation partially depends on the downregulation of HSP70 and HSP90 in L-02 hepatocytes (Xiao et al., 2012). Moreover, HSP69s and HSP20 mediate ROS through caspase 7 and caspase 9 to further inhibit apoptosis in *Plutella xylostella* sperm and ovary cells (Zhang L. J. et al., 2017).

In addition to HSPs acting directly on the apoptosis pathway, HSP70-enhanced miR-23a prevents NOXA (Bcl-2 family proteins), leading to Bax (Bcl-2 family proteins) activation and cytochrome c releasing from the mitochondria, thereby preventing heat-induced apoptosis in cell culture (Roufayel and Kadry, 2019). The HSP90–Akt–ASK1 complex decreases H_2_O_2_-induced ASK1–p38/JNK signaling and cell apoptosis in human umbilical vein endothelial cells (Zhang et al., 2005). By contrast, HSF1 overexpression can trigger apoptotic cell death programs in a Tdag-51-dependent manner (T-cell death-related genes) (Barna et al., 2018). Under heat stress, HSF1 directly activates Tdag51-related regulatory regions to activate its transcription in HeLa cells (Takaki et al., 2006). However, several studies have reported that TDAG51 may have both pro- and anti-apoptotic functions depending on the cellular context and circumstances. With regard to the pro-apoptotic function of TDAG51, available evidence supports the notion that TDAG51 expression is closely associated with enhanced apoptosis (Park et al., 1996; Lama et al., 2003). With regard to the anti-apoptotic function of TDAG51, TDAG51 may be involved in modulating the expression of antioxidant enzymes or non-enzymatic components to eliminate excess ROS generation as a response to oxidative stress, thereby reducing apoptosis in mouse embryonic fibroblasts (MEFs) (Park et al., 2013). Further studies must address the regulatory mechanisms of the physiological importance of TDAG51 expression in oxidative stress response.

**FIGURE 1:**
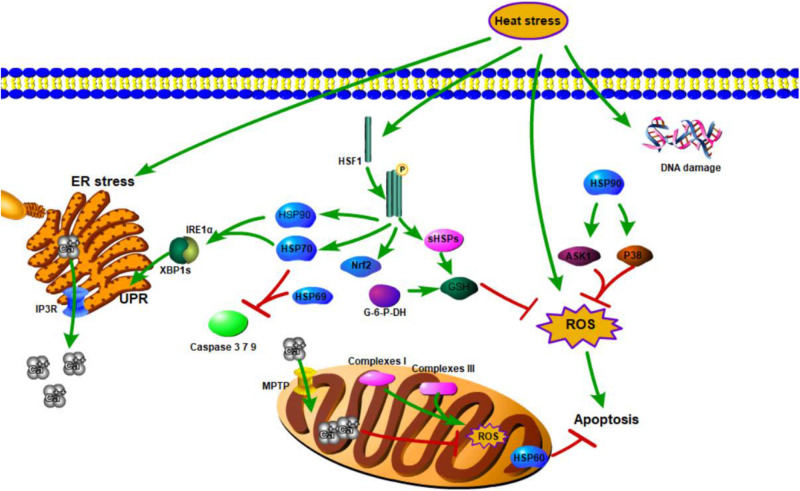
Genetic mechanisms of animal responses to heat.

No direct evidence supports the supposition that calcium mediates TDAG51 under heat stress. However, in mesenchymal transition (EMT) response (during embryonic development in which cells lose epithelial characteristics and gain mesenchymal properties), calcium imbalance can mediate TDAG51 upregulation and prime the cells for mesenchymal transformation in human renal cubular epithelial cells (HRPTECs) (Carlisle et al., 2012), suggesting that calcium may regulate the expression of Tdag51 in the same manner under heat stress. Investigations on the effects of calcium on the apoptotic pathway showed that heat stress may induce apoptosis through the calcium-mediated mitochondrial apoptosis pathway, such as IP3R-regulated cytoplasmic calcium elevation, which further affects Apaf-1, caspase 9, and caspase 3 activations, thereby mediating apoptosis in rabbit corneal cells (Hsu et al., 2011). In addition, the redox sensitivity of IP3R and mitochondria permeability transition pore (MPTP) increases ER calcium releasing and mitochondrial calcium loading (Bhandary et al., 2012). A brief summary of studies on complex regulatory mechanisms, such as oxidative stress, calcium signaling, UPR, or their combinations produced by organisms under thermal stress, is shown in Figure 1. The regulatory effects of HSF1 and calcium under heat stress are summarized in Table 1.

**TABLE 1:** Regulatory effects of heat shock factor 1 (HSF1) and Calcium (Ca) under heat stress.

	**Oxidative stress**	**ER stress**	**Apoptotic pathway**
HSF1	Synergized with nuclear factor erythroid 2-related factor 2 (Nrf2) to regulate sequestosome-1 (SQSTM1)/p62, heme oxygenase-1 (HMOX1), activating transcription factor 3 (Atf3), heat-shock protein family (Hsp)70 and Hsp25/27		Over-expressed HSF1 triggered apoptosis in a Tdag-51-dependent manner
Hsp90	Hsp90 inhibited hydrogen peroxide (H_2_O_2_)-induced ASK1–p38 activation	Interact with inositol-requiring protein 1α (IRE1α) to regulate unfolded protein response (UPR)	Hsp90–Akt–ASK1 complex decreased cell apoptosis
Hsp70	Abolished heat-induced reactive oxygen species (ROS) production, but the mechanism was unknown	Interact with IRE1α to regulate UPR	HSP70-enhanced miR-23a prevented the release of cytochrome c
Small Hsps (sHsps)	Increased glutathione (GSH) and G6PD contributed to reduced state; abolished ROS by TNFα		Interrupted apoptosis by preventing the release of cytochrome c
Calcium	Reduced ROS by regulating complexes I and III	Leaked from the endoplasmic reticulum (ER) into the cytoplasm, which causes ER to induce apoptosis	Cytoplasmic calcium elevation activated Apaf-1, caspase 9 and caspase 3

### Heat Stress and Heat Acclimation

Understanding the survival mechanisms and mitigation strategies of organisms to heat stress is important. Heat acclimation accelerates the rate and alters the magnitude of HSPs expression upon stress application. Under normal conditions, a higher HSP70 level is observed in heat-acclimated organisms than in the non-acclimated rat heart (Horowitz et al., 1997). Compared with the normal group, there are larger HSP reserves in acclimation rats in normal environment, which may contribute to delayed thermal injury (Maloyan et al., 1999). An elevated HSP level may involve a pathway different from that involved in heat stress. Studies on the effects of HSP family on heat acclimation showed that HSP70, HSP90, and HSP110 are required for immediate fish survival at high temperatures, whereas HSP60, HSP70, and HSP78 are needed for their long-term survival at high temperatures (Purohit et al., 2014). mRNA expression of HSP70 from *Cnaphalocrocis medinalis* increases in all five generations of heat selection, but HSP90 increases only in the first two generations in rice leaf folder larvae (Gu et al., 2019). These results indirectly prove the point and also support the premise that HSP70 plays the most important role in HSR and heat acclimation.

## Epigenetic Mechanisms of Animal Responses to Heat Stress and Heat Acclimation

**FIGURE 2:**
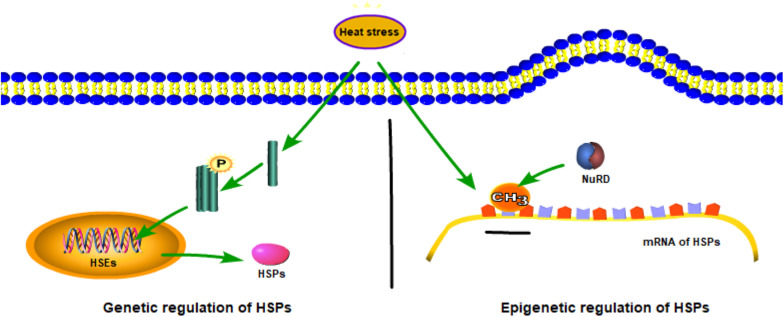
Genetic and epigenetic mechanisms of heat-shock protein family (HSP) synthesis.

Epigenetic regulation changes the gene expression or expression speed through DNA methylation, histone modifications, and other regulatory pathways. In this discussion, HSP is taken as an example (Figure 2). In the genetic mechanism of HSP synthesis, HSF1 becomes trimerized and phosphorylated under heat stress and binds to HSR DNA elements in the nucleus, thereby, activating the transcriptional expression of HSP (Kukreti et al., 2013). By contrast, in the epigenetic regulation of HSP (taken HSP70 as an example), the HSP70 promoter is hypermethylated after acclimation to high temperatures by a reduction in both POU class 2 homeobox 1 (POU2F1, also known as octamer-binding transcription factor-1, is a ubiquitous transcription factor that plays a key role in the regulation of genes related to inflammation and cell cycles) binding and recruitment of the nucleosome remodeling deacetylase (NuRD, combining both deacetylase and remodeling enzymatic activities in a single macromolecular complex) chromatin-remodeling complex, thereby affecting HSP70 expression (Matsuda et al., 2006). In addition to this, epigenetic regulation has been studied through different modifications under environmental stresses. Then, we will discuss the DNA methylation and histone methylation under heat stress.

### Epigenetic Mechanisms of DNA Methylation Under Heat Stress

DNA methylation is a way of epigenetic modification that regulates gene expression. In general, methylation occurs in the promoter region and blocks protein binding, thereby, inhibiting gene transcription (Kuroda et al., 2009). Methylation of the promoter regions of HSP 90 alpha, HSP 90 beta, and HSP 70 is negatively associated with their mRNA expressions in control and heat-treated Naked Neck chicken (Vinoth et al., 2018). However, such an inverse relationship under heat stress cannot be detected in Punjab broiler-2 chicken (Vinoth et al., 2018). Therefore, establishing the link between methylation and gene expression is difficult.

Aside from regulating gene expression, methylation can also form epigenetic memory. DNA methylation is a dynamic process during development and cell differentiation, and several DNA methylation patterns may remain in the form of epigenetic memory (Kim and Costello, 2017). Epigenetic regulation of HSP70 expression via alterations in the CpG methylation profile of a distal promoter region that affects POU2F1 recruitment and H3 deacetylation may reflect heat stress-related epigenetic memory (Kisliouk et al., 2017). Moreover, DNA methylation is a stable epigenetic mark that can be inherited through multiple cell divisions rather than through germline or germ cells (Dor and Cedar, 2018).

### Epigenetic Mechanisms of Histone Methylation Under Heat Stress

Apart from DNA methylation, histone modifications can also affect the transcriptional expression of individual genes by affecting chromosomal domains (Suzuki and Bird, 2008). Studies have focused on the histone modifications of H2B and H3 (Matsuda et al., 2006). The H2B methylation amino acid residue has been identified as methylproline at the N-terminus of H2B (Desrosiers and Tanguay, 1988). Heat shock not only increases the level of H2B methylation but also decreases the level of H3 methylation. Several specific methylated amino acid residues of H3, such as H3 at lysine 4 (H3K4) and H3 at lysine 9 (H3K9), have been identified in rat astrocyte and cortical neuronal cultures (Marinova et al., 2011). H3K4 methylation is correlated with activation of gene transcription, whereas H3K9 methylation is linked to gene repression (Marinova et al., 2011). At the same time, H3 methylation can be inherited. Analysis of the expression profile of *Caenorhabditis elegans* showed that temperature-induced expression of endogenously inhibited repeats can be inherited for multiple generations through the trimethylation of histone H3 lysine 9 (Klosin et al., 2017).

### Epigenetic Mechanisms of Heat Acclimation

The molecular program is the key to the occurrence of heat acclimation. In heat acclimation, accumulating evidence indicates that epigenetic mechanisms are powerful participants in these processes. Epigenetic mechanisms affect DNA accessibility to transcription factors, thereby regulating gene expression and controlling the phenotype. Another important group of epigenetic markers involved in epigenetic mechanisms is miRNAs with miR-297 upregulation in the heat-acclimated *Rattus norvegicus* (Horowitz, 2014; Tetievsky et al., 2014). In addition, decreased miR-1 and miR-206 levels promote HSP70 synthesis (Kwon et al., 2005; Kukreti et al., 2013; Radom-Aizik et al., 2013). In addition to methylation modification, the other posttranslational modifications (phosphorylation and acetylation) in histones play an important role in cytoprotective acclimatory memory. After studying the heat shock element (HSE) binding site of the promoters of HSP70 and HSP90, early histone H3 phosphorylation contributes to histone H4 acetylation, then the constitutively acetylated histone H4 and preserved euchromatin state lead to acclimatory memory (Tetievsky and Horowitz, 2010).

Studies on oxidative phosphorylation have shown that metabolic rate is a major indicator of heat acclimation (Rolfe and Brown, 1997; Seebacher et al., 2009, 2010). Under different conditions of heat stress and heat acclimation, numerous indicators, such as the metabolism of an organism, are widely different. Studies on metabolic capacity have demonstrated that cytochrome c oxidase (COX-respiratory complex IV) sensitivity/activation increases under heat stress (Seebacher, 2009; Seebacher et al., 2009). These results were consistent with a preference of cells for OXPHOS under heat stress (Sajjanar et al., 2019). In contrast, the expression of cytochrome c oxidase, citrate synthase, and proliferator-activated receptor γ coactivator 1α-a (PGC-1α) decreases in heat-acclimated organisms (Horowitz, 2014). Combined with larger HSP reserves in acclimation organism discussed above and that HSP70 was found to inhibit OXPHOS, we make a hypothesis that HSP70 inhibits OXPHOS in the acclimation organism and simultaneously enhances glycolysis to compensate the ATP unbalance (Wang et al., 2012). The OXPHOS complex exhibits different regulatory mechanisms under heat stress and heat acclimation. Another explanation states that heat acclimation reduces the thermal sensitivity of respiration of freshwater and marine animals to temperature changes that, in turn, decreases mitochondrial energy metabolism (Seebacher et al., 2014).

Another hallmark of heat acclimation is high mitochondrial calcium content (Assayag et al., 2012). Calcium content, metabolic rate, and ROS levels are closely related (Sohal and Allen, 1985; Cairns, 2009). Existing evidence shows that different mitochondrial metabolic states cause calcium to induce different effects on mitochondrial ROS levels (Adam-Vizi and Starkov, 2010). Under heat stress, calcium homeostasis is mediated by the chloride intracellular channels (CLICs) through the ryanodine receptor gene (RYR) in the ER membrane, whereas both the CLIC2 and RYR genes are identified as differentially methylated genes (Board et al., 2004). Aside from this pathway, methylated pyruvate kinase M2 (PKM2) isoforms can also alter the influx of calcium from the ER to the mitochondria by suppressing IP3R in tumors in MDA-MB-231 cells (Liu et al., 2017). Here, we make a hypothesis that DNA methylation may regulate the transport of calcium in various organs by methylating specific genes, thereby contributing to the development of heat acclimation.

## Limitation

Although numerous studies have been conducted on the genetic and epigenetic mechanisms involved in HSR, several issues and questions remain to be elucidated. Many members of the HSP family play different roles. The current researches on HSP70 are relatively comprehensive, and the research of other members is relatively scarce. In addition, the experimental conditions set by these studies were different. They performed either *in vivo* or *in vitro* experiments, used different cell cultures, and utilized different organs of different species. It is hard to form a complete network regulation structure, and more experiments need to be supplemented and studied on the model organisms. Heat acclimation is a survival mechanism in organisms. At present, it is more based on the length of time to divide the heat acclimation into short- (<7 days), medium- (8–14 days), and long-term (>15 days) acclimation, which were summarized by Garrett et al. (2011). At present, many studies have shown that many genes are related to heat acclimation, but there is no clear statement, such as which genes or pathways play a key role in it during the formation of heat acclimation. For calcium, its main role is to change the microenvironment of cells by flowing between ER, cytoplasm, and mitochondria. Calcium plays an auxiliary role in oxidative stress, ER stress, and apoptosis, but a clear regulatory mechanism still needs a lot of experimental proof, such as affecting three-dimensional conformation or releasing responsive proteins. In addition, other components, such as miRNAs, have not been extensively investigated.

## Conclusion

Heat greatly affects animal metabolism. Several heat sensors, including HSF1 and calcium, have been reported. As HSF1 production, HSPs facilitate the synthesis and ensure the structural stability of other intracellular proteins. The primary role of HSPs is to improve heat acclimation and form long-term memory. These two biological processes may be two separate processes and have different requirements for HSP production. Calcium, as a secondary messenger, functions by flowing between the ER, cytoplasm, and the mitochondria and is regulated by IP3R and MPTP or methylated genes, such as CLIC2, RYRs, and PKM2. Under heat stress, HSPs in the mitochondria and cytoplasm protect damaged proteins, reduce ROS in cells, and change metabolic processes, thereby further promoting the formation of heat acclimation. HSPs are the common regulatory pathway between immunity stress and heat acclimation. Immunity stress enables organisms to resist heat stress, whereas heat acclimation allows them to adapt to their environment. Further researches should also focus on specific genetic and epigenetic regulatory mechanisms involved in animal thermal response.

## Author Contributions

CL, WZ, and JW wrote the manuscript. CL and WZ contributed to the reagents, materials, and analysis tools. All authors contributed to the article and approved the submitted version.

## Conflict of Interest

The authors declare that the research was conducted in the absence of any commercial or financial relationships that could be construed as a potential conflict of interest.
